# IRF-8 regulates expansion of myeloid-derived suppressor cells and Foxp3^+^ regulatory T cells and modulates Th2 immune responses to gastrointestinal nematode infection

**DOI:** 10.1371/journal.ppat.1006647

**Published:** 2017-10-02

**Authors:** Rajesh M. Valanparambil, Mifong Tam, Pierre-Paul Gros, Jean-Philippe Auger, Mariela Segura, Philippe Gros, Armando Jardim, Timothy G. Geary, Keiko Ozato, Mary M. Stevenson

**Affiliations:** 1 Division of Experimental Medicine, Department of Medicine, McGill University, Montreal, Quebec, Canada; 2 The Research Institute of the McGill University Health Centre, Montreal, Quebec, Canada; 3 Centre for Host-Parasite Interactions, Institute of Parasitology, McGill University, Ste-Anne de Bellevue, Quebec, Canada; 4 Department of Pathology and Microbiology, Faculty of Veterinary Medicine, University of Montreal, St. Hyacinthe, Quebec, Canada; 5 Department of Biochemistry, McGill University, Montreal, Quebec, Canada; 6 Division of Developmental Biology, National Institute of Child Health and Human Development, NIH, Bethesda MD, United States of America; 7 Department of Microbiology and Immunology, McGill University, Montreal, Quebec, Canada; New York University, UNITED STATES

## Abstract

Interferon regulatory factor-8 (IRF-8) is critical for Th1 cell differentiation and negatively regulates myeloid cell development including myeloid-derived suppressor cells (MDSC). MDSC expand during infection with various pathogens including the gastrointestinal (GI) nematode *Heligmosomoides polygyrus bakeri* (Hpb). We investigated if IRF-8 contributes to Th2 immunity to Hpb infection. *Irf8* expression was down-regulated in MDSC from Hpb-infected C57BL/6 (B6) mice. IRF-8 deficient *Irf8*^*-/-*^ and BXH-2 mice had significantly higher adult worm burdens than B6 mice after primary or challenge Hpb infection. During primary infection, MDSC expanded to a significantly greater extent in mesenteric lymph nodes (MLN) and spleens of *Irf8*^*-/-*^ and BXH-2 than B6 mice. CD4^+^GATA3^+^ T cells numbers were comparable in MLN of infected B6 and IRF-8 deficient mice, but MLN cells from infected IRF-8 deficient mice secreted significantly less parasite-specific IL-4 ex vivo. The numbers of alternatively activated macrophages in MLN and serum levels of Hpb-specific IgG1 and IgE were also significantly less in infected *Irf8*^*-/-*^ than B6 mice. The frequencies of antigen-experienced CD4^+^CD11a^hi^CD49d^hi^ cells that were CD44^hi^CD62L^-^ were similar in MLN of infected *Irf8*^*-/-*^ and B6 mice, but the proportions of CD4^+^GATA3^+^ and CD4^+^IL-4^+^ T cells were lower in infected *Irf8*^*-/-*^ mice. CD11b^+^Gr1^+^ cells from naïve or infected *Irf8*^*-/-*^ mice suppressed CD4^+^ T cell proliferation and parasite-specific IL-4 secretion in vitro albeit less efficiently than B6 mice. Surprisingly, there were significantly more CD4^+^ T cells in infected *Irf8*^*-/-*^ mice, with a higher frequency of CD4^+^CD25^+^Foxp3^+^ T (Tregs) cells and significantly higher numbers of Tregs than B6 mice. In vivo depletion of MDSC and/or Tregs in *Irf8*^*-/-*^ mice did not affect adult worm burdens, but Treg depletion resulted in higher egg production and enhanced parasite-specific IL-5, IL-13, and IL-6 secretion ex vivo. Our data thus provide a previously unrecognized role for IRF-8 in Th2 immunity to a GI nematode.

## Author summary

We investigated if IRF-8, which is critical for Th1 immunity and negatively regulates myeloid cell development including MDSC, contributes to Th2 immunity to the gastrointestinal nematode *Heligmosomoides polygyrus bakeri* (Hpb). *Irf8* expression was down-regulated in MDSC from infected C57BL/6 (B6) mice. Hpb-infected IRF-8 deficient mice had significantly higher adult worm burdens than B6 mice. There were significantly more MDSC, fewer alternatively activated macrophages, lower serum levels of Hpb-specific antibodies in infected IRF-8 deficient than B6 mice, and MLN cells from infected IRF-8 deficient mice secreted less parasite-specific IL-4 ex vivo. There were similar frequencies of antigen-experienced CD4^+^CD11a^hi^CD49d^hi^ T cells in MLN that were CD44^hi^CD62L^-^ in infected *Irf8*^*-/-*^ and B6 mice, but lower proportions of CD4^+^GATA3^+^ and CD4^+^IL-4^+^ T cells in *Irf8*^*-/-*^ mice. Infected *Irf8*^*-/-*^ mice had a higher frequency of CD4^+^Foxp3^+^ T (Tregs) cells and significantly higher numbers of Tregs compared to infected B6 mice. MDSC from infected *Irf8*^*-/-*^ mice suppressed CD4^+^ T cell effector functions in vitro albeit less efficiently than B6 mice. Treg and/or MDSC depletion did not affect adult worm burdens in infected *Irf8*^*-/-*^ mice, but Treg depletion partially restored Th2 cytokine responses. These data highlight the importance of IRF-8 in Th2 immunity to Hpb infection.

## Introduction

Interferon regulatory factor (IRF)-8 is a member of the IRF family of transcription factors and plays an important role in regulating proinflammatory cytokines especially IL-12p40, which is critical for Th1 cell differentiation [1]. IRF-8 is essential for the development of various myeloid-derived cells including macrophages, dendritic cells (DC), eosinophils, and basophils, but negatively regulates neutrophil differentiation [2, 3]. Through its IRF-8 association domain (IAD), IRF-8 interacts with other transcription factors, such as PU-1, IRF-1, IRF-4, and IRF-2, and plays an important role in immunity against tumors and infections with intracellular pathogens, including bacteria, viruses, and protozoan parasites [4–6]. *Irf8*^*-/-*^ mice develop a disease similar to chronic myeloid leukemia characterized by expansion of immature Gr1^+^ granulocytes [7]. Partial or total loss-of-function of IRF-8 results in decreased resistance to infections with intracellular pathogens such as *Toxoplasma gondii* in mice and *Mycobacterium tuberculosis* in humans [8, 9].

BXH-2 mice, a recombinant inbred strain generated by a cross between C57BL/6 (B6) and C3H/HeJ mice, carry an arginine-to-cysteine substitution at position 294 in the IAD of the *Irf8* gene [10, 11]. In the presence of this mutation, IRF-8 is unable to bind to its partner transcription factors resulting in a phenotype similar to *Irf8*^*-/-*^ mice. BXH-2 mice display increased myeloproliferation of CD11b^+^Gr1^+^ cells with splenomegaly and lymph node enlargement [10, 12]. Like *Irf8*^*-/-*^ mice, BXH-2 mice are highly susceptible to infections with intracellular pathogens including *M*. *bovis* (BCG), *Salmonella enterica* serovar *Typhimurium*, and *Plasmodium chabaudi* AS as well as *M*. *tuberculosis* [13, 14].

During infection, IRF-8 is induced in antigen presenting cells (APC) by IFN-γ and TLR ligands, binds to IRF-4, and stimulates IL-12 as well as IL-18 secretion, resulting in CD4^+^ Th1 cell differentiation and elimination of intracellular pathogens [15, 16]. The severe immunodeficiency and failure to produce IFN-γ and Th1-associated cytokines evident in *Irf8*^*-/-*^ and BXH-2 mice is considered to be due to a deficiency in CD11c^+^CD8α^+^ DC responsible for producing high levels of IL-12 as well as type 1 interferon [12, 17]. On one hand, *Irf8*^*-/-*^ mice are resistant to experimental autoimmune encephalomyelitis (EAE) due to a deficiency in Th17 cell expansion [18]. On the other hand, Ouyang et al observed that IRF-8 deficient mice have enhanced Th17 cell differentiation in vitro and adoptive transfer of naïve CD4^+^ T cells from *Irf8*^*-/-*^ mice results in enhanced Th17 responses and more severe intestinal inflammation in a Rag^-/-^ mouse model of colitis than cells from control mice [19]. These data highlight the importance of IRF-8 in Th1- and Th17-mediated immune responses, but the effect of IRF-8 deficiency on Th17 cell differentiation appears to be variable depending on the experimental model used.

Earlier, Giese *et al* observed that *Irf8*^*-/-*^ mice are susceptible to *Leishmania major* infection compared to wild-type (WT) B6 mice, which are resistant [15]. Draining lymph node and spleen cells from infected *Irf8*^*-/-*^ mice produce high levels of antigen-specific IL-4, the signature Th2 cytokine, and little or no IFN-γ, compared to cells from infected B6 mice. Based on these findings, it was concluded that a deficiency in IRF-8 leads to Th2 cell differentiation as a default pathway due to deficient IL-12 secretion by APC [15]. These data indicate that IRF-8 is critical for Th1- and Th17-mediated immune responses, but its role in Th2 responses has not been investigated.

*Heligmosomoides polygyrus bakeri* (Hpb) is a natural pathogen of mice that serves as a laboratory model of gastrointestinal (GI) nematode infection [20]. Hpb infection is associated with strong immunosuppressive effects and induces regulatory T cells (Tregs), tolerogenic DC, regulatory B cells, and high levels of the immunoregulatory cytokines IL-10 and TGFβ [20–23]. Primary Hpb infection is chronic and can last up to several months in some inbred mouse strains, including B6 mice [21]. Adult worms are rapidly cleared after challenge infection due to a highly polarized Th2 immune response critically dependent on IL-4-producing CD4^+^GATA3^+^ T cells, alternatively activated macrophages (AAMØ), B cells, and parasite-specific IgG1 [22]. Together, these cells and their effector molecules play important roles in eliminating the infection.

Recently, we showed that F4/80^-^CD11b^+^Gr1^hi^ myeloid-derived suppressor cells (MDSC) increase dramatically during primary Hpb infection in B6 mice [24]. We also demonstrated that MDSC contribute to chronic infection characteristic of these hosts by suppressing parasite-specific Th2 responses. MDSC are a heterogeneous population of immature myeloid cells that suppress effector CD4^+^ and CD8^+^ T cell responses, including proliferation and cytokine production [25]. Interestingly, MDSC are present in high numbers in naïve *Irf8*^*-/-*^ mice [26]. MDSC expansion is associated with faster tumor growth in *Irf8*^*-/-*^ mice than in WT B6 mice, while expansion of MDSC is diminished during tumor growth in transgenic mice that over-express *Irf8*. The expression of *Irf8* is down-regulated in breast cancer patients, and the level correlates inversely with the frequency of peripheral MDSC. Together, these data indicate IRF-8 negatively regulate MDSC.

Here, we investigated the contribution of IRF-8 to the development of Th2 immunity to Hpb infection. *Irf8* expression was down-regulated in B6 MDSC after primary Hpb infection, suggesting a role for this transcription factor in regulating Th2 immune responses. IRF-8 deficient *Irf8*^*-/-*^ and BXH-2 mice harboured significantly higher adult worm burdens than B6 mice after primary or challenge Hpb infection. During primary infection, high worm burdens in IRF-8-deficient mice were associated with marked expansion of MDSC in mesenteric lymph node (MLN) and spleen. MLN and spleen cells from infected *Irf8*^*-/-*^ mice secreted significantly lower levels of parasite-specific IL-4 ex vivo than infected B6 mice. Further analysis revealed that deficient IL-4 secretion by cells from infected *Irf8*^*-/-*^ mice was not due to differentiation of naïve CD4^+^ T cells to Th1/Th17 cells. Importantly, lower proportions of CD4^+^ T cells expressed GATA3 and IL-4, but a higher proportion of CD4^+^ T were Foxp3^+^ in *Irf8*^*-/-*^ compared to B6 mice during Hpb infection. Indeed, MDSC from naïve and infected *Irf8*^*-/-*^ mice suppressed antigen-specific IL-4 secretion in vitro. Administration of 5-FU to infected *Irf8*^*-/-*^ mice significantly decreased MDSC in MLN, but there were no effects on the adult worm burden or egg production. We also observed significantly higher numbers of total CD4^+^ T cells and CD4^+^CD25^+^Foxp3^+^ cells in *Irf8*^*-/-*^ mice compared to B6 mice during Hpb infection. Depletion of Tregs in infected *Irf8*^*-/-*^ mice enhanced Th2 cytokine responses without affecting the adult worm burden but egg production was significantly increased.

## Results

### *Irf8* expression is down-regulated in MDSC during Hpb infection and contributes to higher adult worm burdens

**Fig 1:**
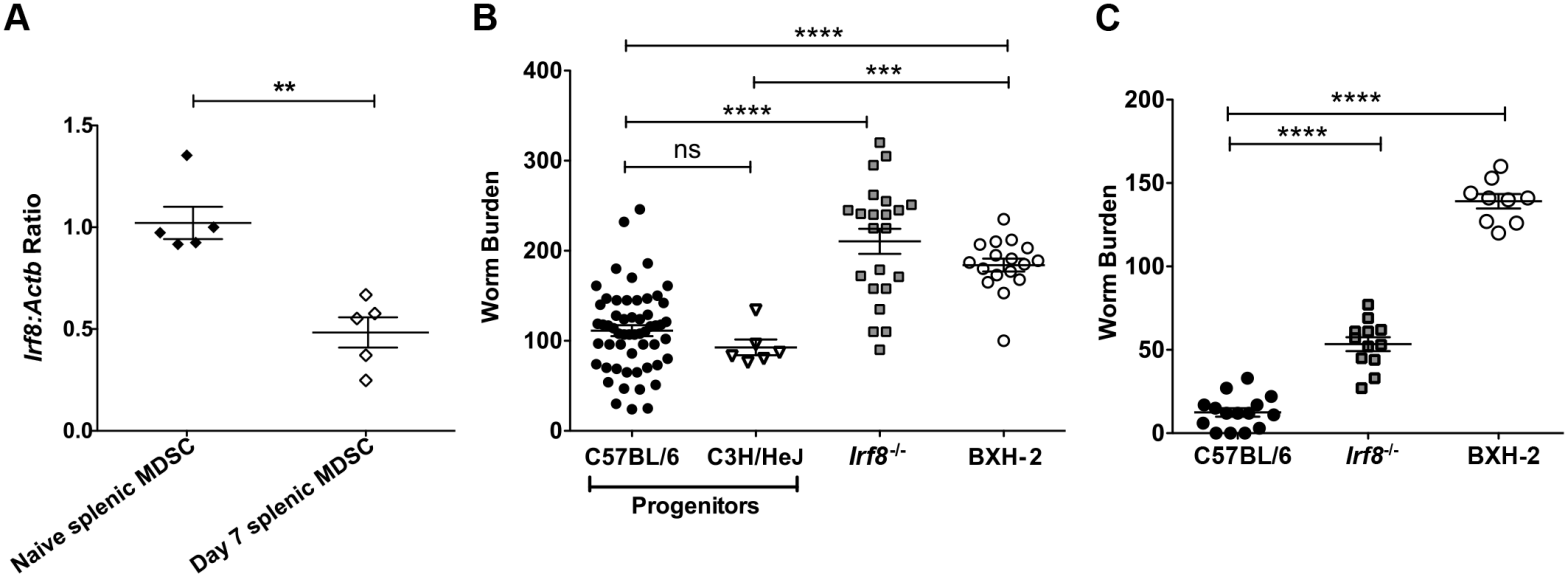
*Irf8* expression is down-regulated in MDSC and contributes to higher worm burdens after Hpb infection. (A) *Irf8* expression in CD11b^+^Gr1^+^ cells purified from spleens of naïve and infected C57BL/6 (B6) mice on day 7 p.i. Each point represents cells pooled from 2 naïve mice or one infected B6 mouse. Representative results of one of two replicate experiments are shown. Data are presented as relative quantity of *Irf8* normalized against the endogenous control *Actb*. (B) Adult worm burden after primary Hpb infection in WT B6 and progenitor C3H/HeJ mice compared to *Irf8*^*-/-*^ and IRF-8 deficient BXH-2 mice. The worm burdens of individual mice pooled from independent experiments are shown. (C) Adult worm burden after challenge Hpb infection in WT B6, *Irf8*^*-/-*^ and BXH-2 mice. The worm burdens of individual mice pooled from independent experiments are shown. Data are presented as mean ± SEM. ns, not significant; **, *p* ≤ 0.01; ***, *p* ≤ 0.001; ****, *p* ≤ 0.0001.

We recently showed that MDSC increase significantly in local and systemic lymphoid tissues in B6 mice during primary Hpb infection and contribute to chronic infection by suppressing Th2 responses [24]. *Irf8* levels are decreased in MDSC recovered from tumor-bearing mice and cancer patients [26]. To determine if *Irf8* expression is modulated during Hpb infection, we analyzed the levels in tissues as well as purified CD11b^+^Gr1^+^ cells from naïve and infected B6 mice by qRT-PCR. There were no differences in *Irf8* expression in MLN or spleen from naïve versus Hpb-infected mice (S1 Fig). However, *Irf8* expression was significantly down-regulated in purified CD11b^+^Gr1^+^ cells from infected compared to naïve B6 mice (Fig 1A).

To investigate if IRF-8 plays a role in Th2 immunity, WT B6 and *Irf8*^*-/-*^ mice were infected with Hpb and the adult worm burden was determined on day 14 after primary infection. A significantly higher number of adult worms was recovered from *Irf8*^*-/-*^ compared to B6 mice (Fig 1B). We confirmed the importance of IRF-8 deficiency in controlling primary Hpb infection by examining the adult worm burden in BXH-2 mice that bear a mutation in the IAD region of the *Irf8* gene [11]. Consistent with our findings in *Irf8*^*-/-*^ mice, BXH-2 mice harboured significantly more adult worms than progenitor B6 or C3H/HeJ mice (Fig 1B). We also determined if a deficiency in IRF-8 perturbed protective Th2 immunity required for eliminating adult worms after challenge Hpb infection. Two weeks after re-infection of anthelmintic-treated mice, *Irf8*^*-/-*^ as well as BXH-2 (Fig 1C) mice had significantly more adult worms in the small intestine than B6 mice. These data indicate that *Irf8* expression is down-regulated during primary Hpb infection and a partial or total loss-of-function in IRF-8 affects immune control of the adult worm burden after both primary and challenge Hpb infections.

### IRF-8 deficiency is associated with expansion of MDSC and decreased CD4^+^ Th2 cell effector function after Hpb infection

To determine if differences in immune cell populations in lymphoid tissues, especially in MLN, contributed to higher worm burdens in IRF-8 deficient mice, we examined the numbers and cellular composition of MLN and spleen from naïve and Hpb-infected B6 versus similar groups of *Irf8*^*-/-*^ and BXH-2 mice. There were significantly higher numbers of total cells in MLN and spleen of naïve *Irf8*^*-/-*^, but not in BXH-2 mice, than B6 mice (S2 Fig). On day 14 after primary or challenge infection, there were marked increases in the total cellularity of lymphoid tissues from B6 as well as IRF-8 deficient mice with significantly higher cell numbers in the MLN and spleen of *Irf8*^*-/-*^ compared to B6 mice.

As previously reported, the number of F4/80^-^CD11b^hi^Gr1^hi^ cells was significantly higher in the spleen of naïve *Irf8*^*-/-*^ compared to B6 mice (S3A Fig) [26]. In addition, we observed a significantly higher frequency and number of these cells in MLN of naïve *Irf8*^*-/-*^ than B6 mice (S3A & S4 Figs). There was also a significantly higher frequency of MDSC in MLN of naïve BXH-2 vs. B6 mice although the number of MDSC was similar (S3B & S4 Figs). Similar to naïve *Irf8*^*-/-*^ mice, there was a significantly higher number of MDSC in the spleen of naïve BXH-2 than B6 mice.

**Fig 2:**
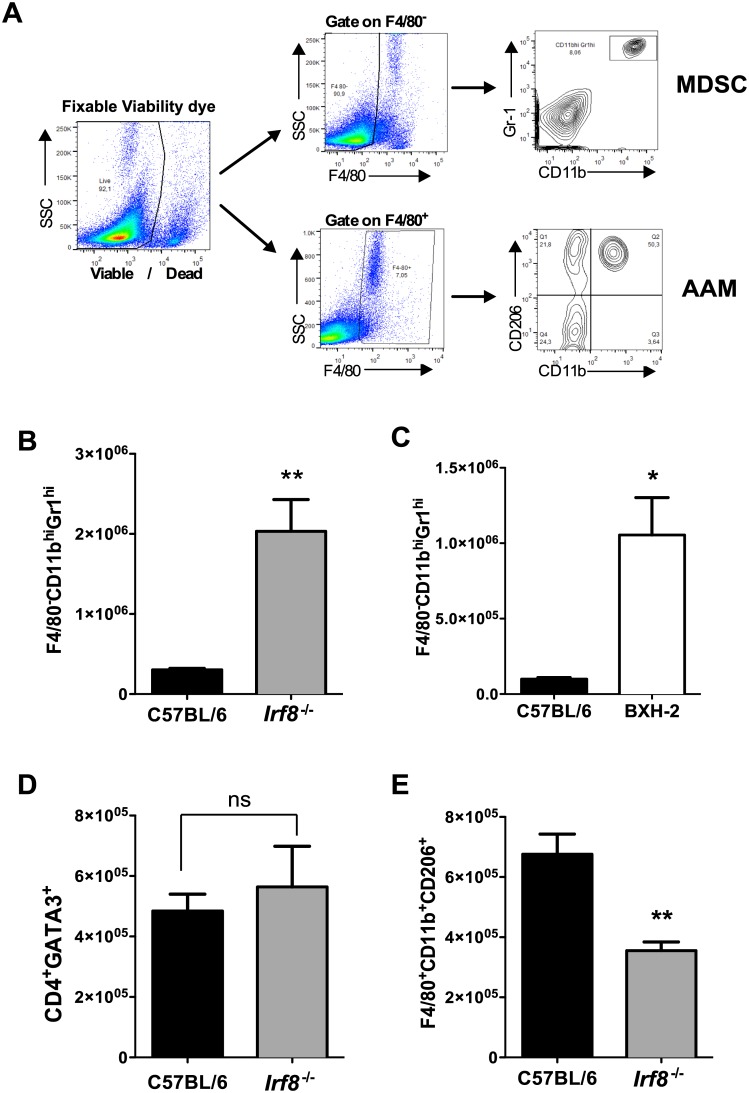
Immunophenotype of cells in MLN of C57BL/6 and IRF-8 deficient mice after primary Hpb infection. (A) Gating strategy used to analyze F4/80^-^CD11b^hi^Gr1^hi^ (MDSC) and F4/80^+^CD11b^+^CD206^+^ (AAMØ) cells by flow cytometry. Total numbers of F4/80^-^CD11b^hi^Gr1^hi^ cells in MLN of (B) C57BL/6 (B6) and *Irf8*^*-/-*^ mice and (C) B6 and BXH-2 mice on day 14 p.i. Total numbers of (D) CD4^+^GATA3^+^ T cells and (E) F4/80^+^CD11b^+^CD206^+^ cells in MLN of B6 and *Irf8*^*-/-*^ mice on day 14 p.i. n = 5 mice/group. Data are representative of three replicate experiments in B6 and *Irf8*^*-/-*^ mice and two replicate experiments in B6 and BXH-2 mice. Data are presented as mean ± SEM. ns, not significant; *, *p* ≤ 0.05; **, *p* ≤ 0.01.

Consistent with our previous observation, the frequency of F4/80^-^CD11b^hi^Gr1^hi^ cells increased significantly in MLN of infected compared to naïve B6 mice on day 14 post infection (p.i.) (S4 Fig) [24]. In contrast, the frequency of MDSC decreased during Hpb infection in IRF-8 deficient mice compared to naïve mice (S4 Fig). Importantly, there were significantly higher numbers of F4/80^-^CD11b^hi^Gr1^hi^ cells in the MLN of infected *Irf8*^*-/-*^ and BXH-2 mice compared to B6 mice (Fig 2A–2C). Interestingly, the numbers of CD4^+^GATA3^+^ T cells in MLN were similar in infected *Irf8*^*-/-*^ and B6 mice (Fig 2D), but the number of F4/80^+^CD11b^+^CD206^+^ AAMØ was significantly lower in infected *Irf8*^*-/-*^ mice (Fig 2E). Similar findings were observed for CD4^+^GATA3^+^ cells and AAMØ in MLN of BXH-2 compared to B6 mice after primary Hpb infection (S3C and S3D Fig).

**Fig 3:**
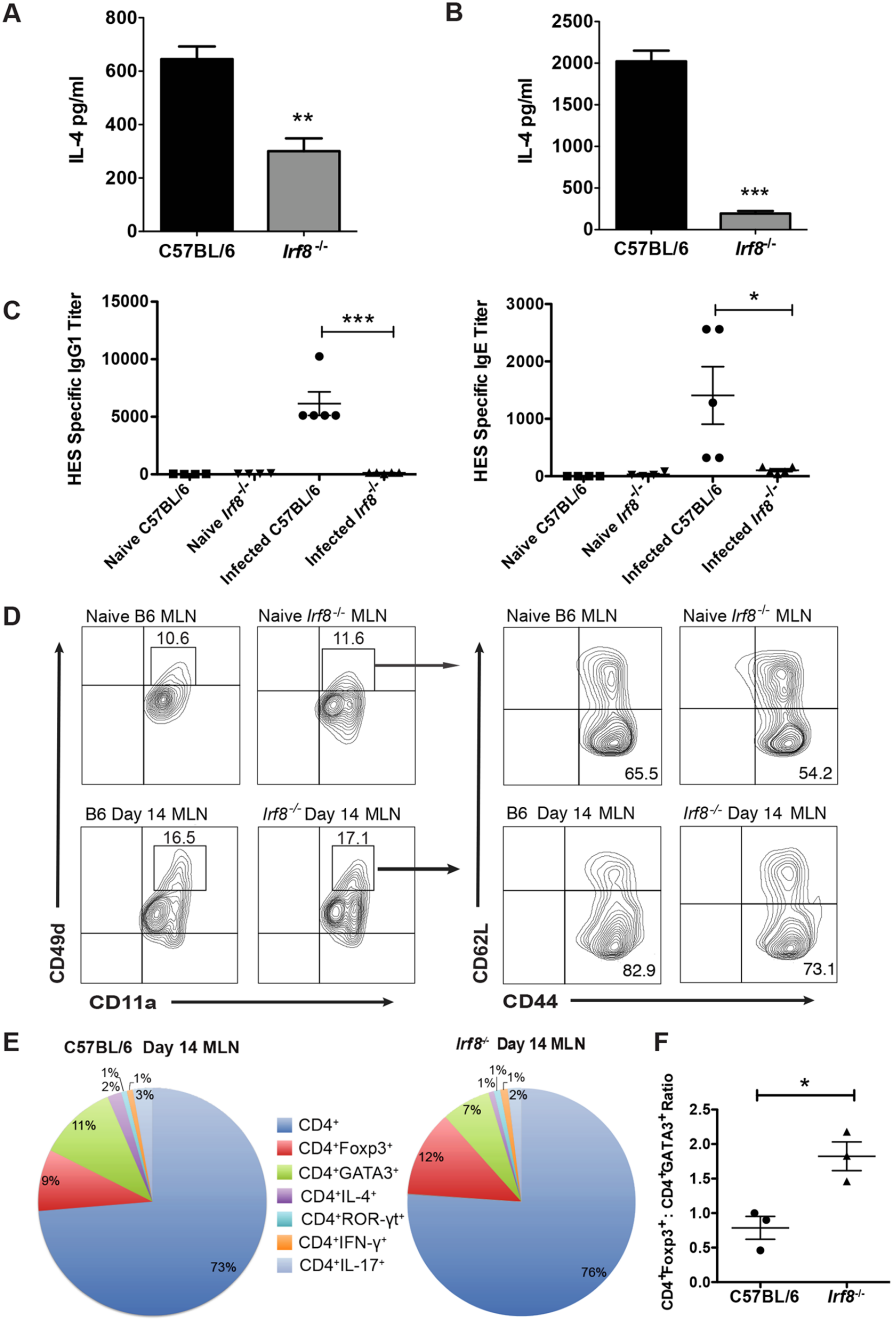
Analysis of CD4^+^ Th2 cell effector function in Hpb-infected C57BL/6 and *Irf8*^*-/-*^ mice. (A) MLN and (B) spleen cells obtained from C57BL/6 (B6) and *Irf8*^*-/-*^ mice (n = 5 mice per group) on day 14 p.i. were stimulated ex vivo with 50 μg AWH. Supernatants were collected 48 h later and IL-4 levels determined by ELISA. Data representative of two to three replicate experiments are presented as mean ± SEM. **; *p* ≤ 0.01; ***, p≤0.001. (C) HES-specific IgG1 and IgE titers in the sera of naïve and infected B6 and *Irf8*^*-/-*^ mice on day 14 p.i. Each point represents one mouse. Mean ± SEM for each group is shown. *, *p* ≤ 0.05; ***, p≤0.001. (D) MLN cells were obtained from naïve and infected B6 or *Irf8*^*-/-*^ mice on day 14 p.i. (n = 3 mice per group). CD11a and CD49d expression were analyzed on gated CD4^+^ T cells by flow cytometry. CD11a^hi^CD49d^hi^ cells were gated and the expression of CD44 and CD62L was analyzed. Representative contour plots of one mouse from each group are shown for co-expression of CD11a and CD49d and CD44 and CD62L. Data presented for B6 mice are from one of 2 replicate experiments performed. (E) Gated CD4^+^ T cells obtained from naïve and infected B6 or *Irf8*^*-/-*^ mice on day 14 p.i. (n = 3 mice/group) were analyzed for intracellular expression of GATA3, Foxp3, IL-4, ROR-γt, IFN-γ, and IL-17 by flow cytometry. The proportions of each cell type are shown as a percent of total CD4^+^ T cells. (F) The ratios of the proportions of CD4^+^Foxp3^+^ to CD4^+^GATA3^+^ T cells were determined for C57BL/6 and *Irf8*^*-/-*^ mice based on the data shown in panel E. Each point represents one mouse. Mean ± SEM for each group is shown. *, *p* ≤ 0.05.

Despite similar numbers of CD4^+^GATA3^+^ T cells in the MLN of Hpb-infected B6 and IRF-8*-*deficient mice, IL-4 secretion ex vivo was significantly lower in response to AWH by MLN as well as spleen cells from infected *Irf8*^*-/-*^ compared to B6 mice (Fig 3A and 3B). Likewise, MLN cells from infected BXH-2 mice produced significantly lower levels of AWH-specific IL-4 ex vivo than infected B6 mice (S3E Fig). In addition, serum levels of HES-specific IgG1 and IgE were significantly lower in Hpb-infected *Irf8*^*-/-*^ compared to B6 mice (Fig 3C).

To further characterize the CD4^+^ T cell response in IRF-8 deficient mice after Hpb infection, we used the surrogate marker approach described by McDermott at al to identify antigen-experienced CD4^+^ T cells [27]. Previous studies in mice infected with a variety of pathogens have validated this approach, which is based on increased co-expression of the integrins CD11a and CD49d, to track CD4^+^ T cell responses even without knowledge of pathogen-specific epitopes [28–30]. As expected, there were low frequencies of CD4^+^CD11a^hi^CD49d^hi^ cells in MLN of naïve B6 and *Irf8*^*-/-*^ mice (Fig 3D upper panels). After Hpb infection, the frequency of this population increased to similar levels in MLN of B6 and *Irf8*^*-/-*^ mice (Fig 3D lower panels). Between 50–60% of CD4^+^CD11a^hi^CD49d^hi^ cells in naïve B6 and *Irf8*^*-/-*^ mice were CD44^hi^CD62L^-^ and the frequency of this population increased in B6 as well as *Irf8*^*-/-*^ mice to 70–80% during Hpb infection.

To address the possibility that CD4^+^ T cells differentiated to Th cell subsets other than Th2 cells in *Irf8*^*-/-*^ mice during Hpb infection, we analyzed the expression of transcription factors and cytokines that distinguish various Th cell subsets, including Tregs, in MLN CD4^+^ T cells. Immunophenotyping of gated CD4^+^ T cells showed that 73% of total CD4^+^ T cells in MLN of B6 mice and 76% in *Irf8*^*-/-*^ mice were undifferentiated on day 14 p.i. (Fig 3E). During Hpb infection, 11% of the total CD4^+^ T cells were GATA3^+^ and 9% were Foxp3^+^ in B6 mice, while 7% expressed GATA3 and 12% expressed Foxp3 in *Irf8*^*-/-*^ mice. There were no significant differences between the proportions of CD4^+^GATA3^+^ and CD4^+^Foxp3^+^ T cells between infected B6 and *Irf8*^*-/-*^ mice, but the ratio of the proportions of Tregs to Th2 cells was significantly higher in infected *Irf8*^*-/-*^ compared to B6 mice (Fig 3F). Consistent with the findings described above that MLN cells from infected *Irf8*^*-/-*^ mice secreted significantly lower levels of parasite-specific IL-4 ex vivo than cells from infected B6 mice, only 1% of CD4^+^ T cells were IL-4^+^ in *Irf8*^*-/-*^ compared to 2% in B6 mice during Hpb infection. The proportions of CD4^+^ T cells that expressed ROR-γt, IL-17 or IFN-γ were low in MLN of both genotypes after infection. These data indicate CD4^+^ T cells in infected *Irf8*^*-/-*^ mice did not differentiate to the Th1 or Th17 pathway as a default, but that Tregs may be the dominate CD4^+^ T cell population in these mice during Hpb infection.

### Suppression of CD4^+^ T cell effector function by MDSC from *Irf8*^*-/-*^ mice

**Fig 4:**
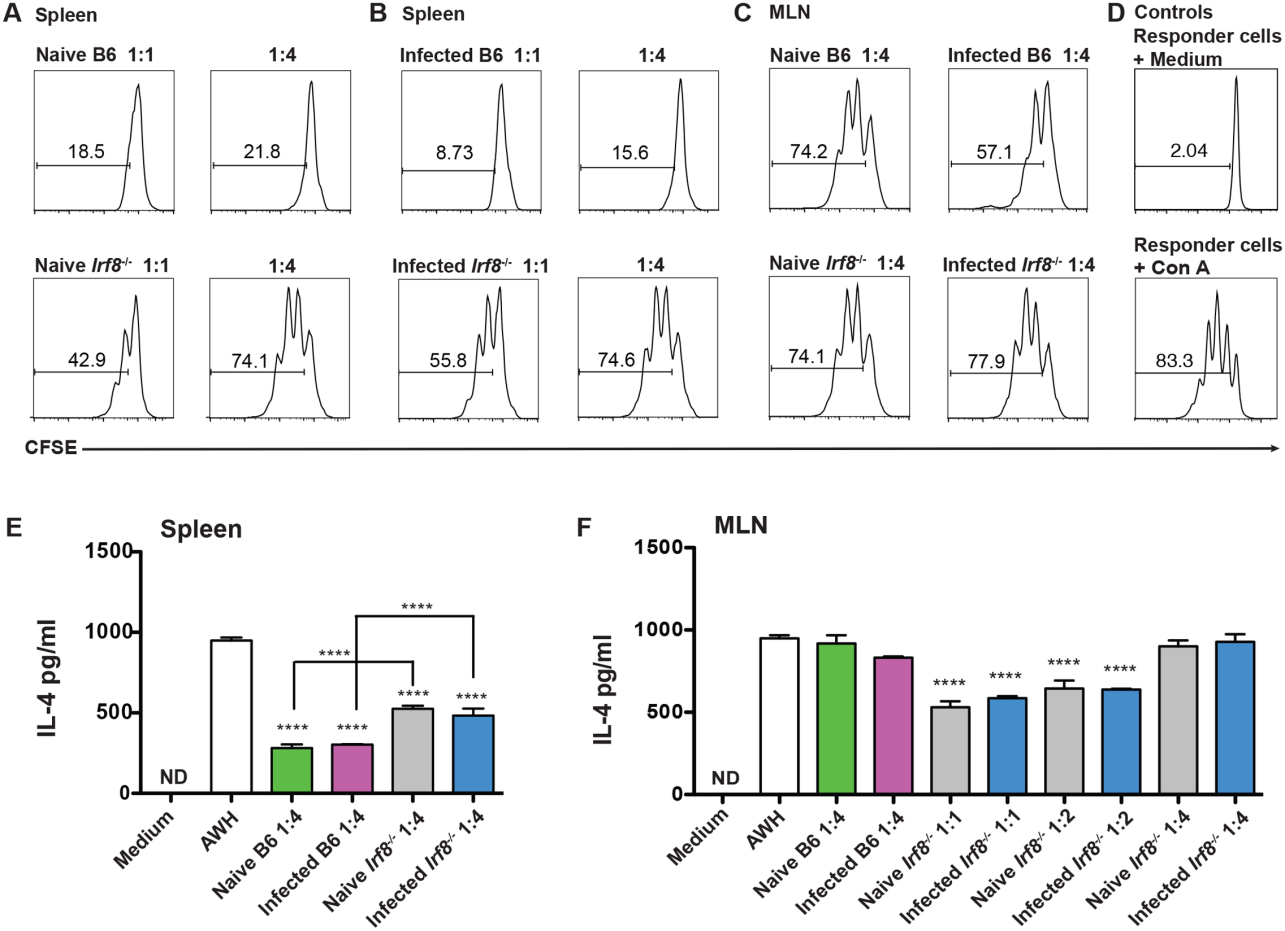
CD4^+^ T cells are suppressed by MDSC from naïve or Hpb-infected C57BL/6 and *Irf8*^*-/-*^ mice. Purified CD11b^+^Gr1^+^ cells from spleen of naïve (A) and infected (B) C57BL/6 (B6) or *Irf8*^*-/-*^ mice were co-cultured with CFSE-labeled spleen cells from naïve B6 mice and stimulated with 2 μg/ml Con A. (C) Purified CD11b^+^Gr1^+^ cells from MLN of naïve or infected C57BL/6 (B6) and *Irf8*^*-/-*^ mice were co-cultured with CFSE-labeled spleen cells from naïve B6 mice and stimulated with 2 μg/ml Con A. (D) Control cultures containing responder cells alone (top) or responder cells stimulated with 2 μg/ml Con A (bottom). CFSE dilution in panels A-D was analyzed in gated CD4^+^ T cells by flow cytometry. The data shown are representative of two replicate experiments. Purified CD11b^+^Gr1^+^ cells from spleens (E) or MLN (F) of naïve or infected B6 and *Irf8*^*-/-*^ mice were co-cultured with spleen cells from infected B6 mice and stimulated with medium or 50 μg AWH. IL-4 levels were determined in 48 h supernatants by ELISA. The ratio of MDSC:responder cells is indicated for each panel. The data shown are representative of two to three replicate experiments and are presented as mean ± SEM. ND, not detectable; ****, *p* ≤ 0.0001.

Previously, we showed that purified CD11b^+^Gr1^+^ cells from Hpb-infected B6 mice potently suppress antigen-specific CD4^+^ T cell proliferation and IL-4 secretion in vitro [24]. To investigate if MDSC from *Irf8*^*-/-*^ mice suppress CD4^+^ T cell proliferation, purified CD11b^+^Gr1^+^ cells from naïve and infected B6 or *Irf8*^*-/-*^ mice were co-cultured with CFSE-labeled spleen cells from naïve B6 mice. Following stimulation with Con A, proliferation was examined in gated CD4^+^ T cells by CFSE staining. On a cell-per-cell basis, CD11b^+^Gr1^+^ cells recovered from the spleens of naïve and infected *Irf8*^*-/-*^ mice were less suppressive than CD11b^+^Gr1^+^ cells from B6 mice at ratios of MDSC to responder cells of 1:1 and 1:4 (Fig 4A and 4B) as well as 1:8 (S5 Fig).

Nevertheless, MDSC from either genotype suppressed CD4^+^ T cell proliferation to Con A in a dose-dependent manner compared to the control (Fig 4D). The low numbers of CD11b^+^Gr1^+^ cells obtained from MLN permitted us to compare the suppressive activity of MDSC from B6 vs. *Irf8*^*-/-*^ mice at only a 1:4 ratio. Although CD11b^+^Gr1^+^ cells purified from MLN of naïve B6 or *Irf8*^*-/-*^ mice suppressed Con A-induced proliferation to a similar albeit negligible extent compared to the control culture, CD11b^+^Gr1^+^ cells from infected B6 mice were more suppressive than cells from infected *Irf8*^*-/-*^ mice (Fig 4C).

We also compared the ability of MDSC from B6 and *Irf8*^*-/-*^ mice to suppress Hpb-specific IL-4 secretion. Purified CD11b^+^Gr1^+^ cells were co-cultured with spleen cells from infected B6 mice and stimulated with AWH. Spleen CD11b^+^Gr1^+^ cells from B6 as well as *Irf8*^*-/-*^ mice, either naïve or infected, significantly suppressed IL-4 secretion compared to spleen cells stimulated with AWH alone (Fig 4E). However, IL-4 secretion was significantly lower in co-cultures with CD11b^+^Gr1^+^ cells from naïve or infected B6 compared to similar groups of *Irf8*^*-/-*^ mice. Importantly, CD11b^+^Gr1^+^ cells purified from the MLN of naïve or infected *Irf8*^*-/-*^ mice significantly suppressed AWH-induced IL-4 secretion at ratios of 1:1 and 1:2 MDSC to responder cells compared to responder cells stimulated with AWH in the absence of MDSC (Fig 4F).

### MDSC depletion does not alter adult worm burdens in infected *Irf8*^*-/-*^ mice

**Fig 5:**
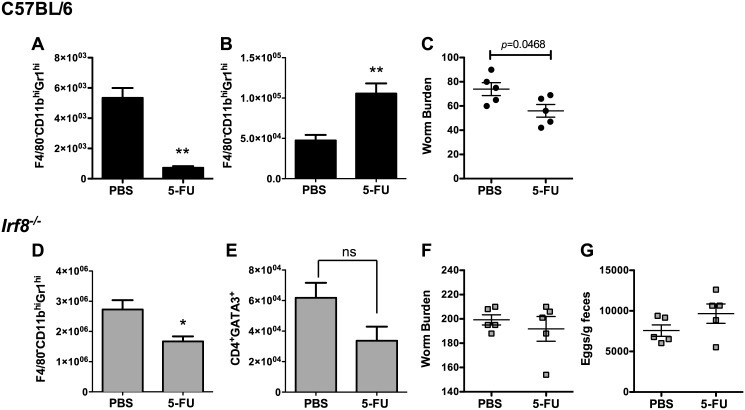
Effect of MDSC depletion on worm burdens in Hpb-infected C57BL/6 and *Irf8*^*-/-*^ mice. Numbers of F4/80^-^CD11b^hi^Gr1^hi^ cells in MLN of infected C57BL/6 (B6) mice treated with PBS or 5-FU on (A) day 7 or (B) day 14 p.i. (C) Adult worm burdens on day 14 p.i. of individual B6 mice treated with PBS or 5-FU. Numbers of (D) F4/80^-^CD11b^hi^Gr1^hi^ and (E) CD4^+^GATA3^+^ T cells in MLN of infected *Irf8*^*-/-*^ mice treated with PBS or 5-FU on day 14 p.i. (F) Adult worm burdens on day 14 p.i. and (G) fecal egg counts on day 13 p.i. of individual infected *Irf8*^*-/-*^ mice treated with PBS or 5-FU. Data are representative of two replicate experiments each with n = 5 mice per group. Data are presented as mean ± SEM. ns, not significant; *, *p* ≤ 0.05; **, *p* ≤ 0.01.

Administration of the chemotherapeutic agent 5-fluorouracil (5-FU) to tumor-bearing mice significantly decreases MDSC in tissue and peripheral blood and restores anti-cancer immune responses [31, 32]. To determine if 5-FU treatment depletes MDSC and enhances Th2 immunity in Hpb-infected mice, B6 mice were treated i.p. with 5-FU or PBS as a control on day -2 prior to infection and on days 5 and 12 p.i. MDSC were significantly decreased in MLN of 5-FU-treated compared to PBS-treated B6 mice on day 7 p.i. (Fig 5A). Consistent with previous findings in tumor-bearing mice treated with anti-Gr-1 mAb to deplete MDSC, MDSC depletion was transient in 5-FU-treated mice and the numbers of MDSC in MLN rebounded and were significantly higher compared to PBS-treated B6 mice on day 14 p.i. (Fig 5B) [33]. Despite this, the adult worm burden was significantly but modestly decreased after 5-FU treatment compared to PBS control (Fig 5C).

To investigate the effects of MDSC depletion in Hpb-infected *Irf8*^*-/-*^ mice, we used a similar protocol to administer 5-FU to these mice. MDSC were significantly reduced in 5-FU treated *Irf8*^*-/-*^ mice compared to PBS control mice on day 14 p.i. (Fig 5D). MDSC depletion did not alter the numbers of CD4^+^GATA3^+^ T cells (Fig 5E), adult worm burden (Fig 5F) or egg production (Fig 5G) in infected *Irf8*^*-/-*^ mice.

### Higher numbers of CD4^+^Foxp3^+^ Tregs are associated with decreased Th2 responses in Hpb-infected *Irf8*^*-/-*^ mice

**Fig 6:**
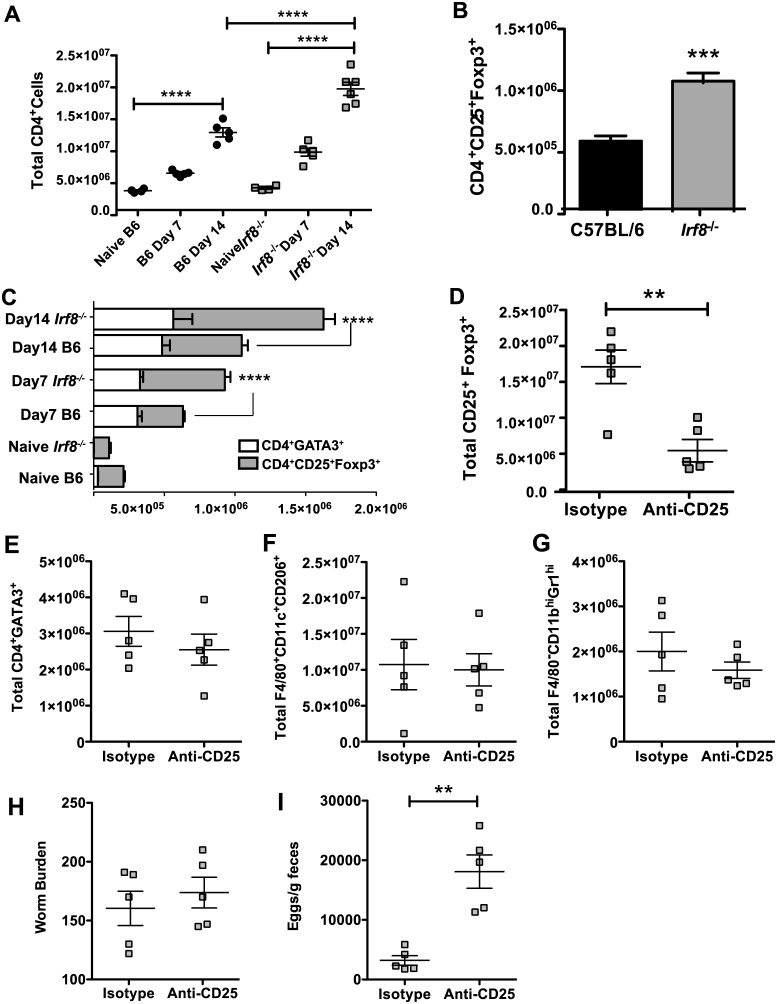
CD4^+^CD25^+^Foxp3^+^ T cell expansion is significantly greater in *Irf8*^*-/-*^ than C57BL/6 mice during Hpb infection. (A) Numbers of total CD4^+^ T cells in MLN of naïve and infected C57BL/6 (B6) or *Irf8*^*-/-*^ mice on days 7 and 14 p.i. (B) Numbers of CD4^+^CD25^+^Foxp3^+^ cells in MLN of infected B6 and *Irf8*^*-/-*.^mice on day 14 p.i. (C) Numbers of CD4^+^GATA3^+^ and CD4^+^CD25^+^Foxp3^+^ T cells in MLN of naïve B6 and *Irf8*^*-/-*.^mice and on days 7 and 14 p.i. (D) Numbers of CD4^+^CD25^+^Foxp3^+^ T cells in MLN of *Irf8*^*-/-*.^mice treated with isotype control antibody or anti-CD25 mAb on day 14 p.i. Numbers of (E) CD4^+^GATA3^+^ T cells, (F) F4/80^+^CD11c^+^CD206^+^ cells, and (G) F4/80^-^CD11b^hi^Gr1^hi^ cells in MLN of *Irf8*^*-/-*.^mice treated with isotype control antibody or anti-CD25 mAb on day 14 p.i. (H) Adult worm burdens on day 14 p.i. and (I) fecal egg counts on day 13 p.i. of *Irf8*^*-/-*^ mice treated with isotype control antibody or anti-CD25 mAb. Data from individual mice (n = 5 mice per group) are presented in panels A and D-I. Data are presented as mean ± SEM. **, *p* ≤ 0.01; ***, *p* ≤ 0.001; ****, *p* ≤ 0.0001.

After Hpb infection, Foxp3^+^ Tregs expand in local and systemic lymphoid tissues but the role of these cells remains ambiguous [34, 35]. Surprisingly, there were significantly higher numbers of total CD4^+^ T cells in MLN of *Irf8*^*-/-*^ than B6 mice on day 14 p.i. (Fig 6A) despite similar numbers of CD4^+^GATA3^+^ T cells in infected mice of both genotypes (Fig 2D). At this time, the number of CD4^+^CD25^+^Foxp3^+^ T cells was significantly higher in infected *Irf8*^*-/-*^ compared to B6 mice (Fig 6B). We performed a kinetic analysis of Th2 versus Tregs in B6 compared to *Irf8*^*-/-*^ mice during infection. The numbers of CD4^+^CD25^+^Foxp3^+^ T cells were similar in naïve B6 and *Irf8*^*-/-*^ mice, but this population expanded early during infection in both B6 and *Irf8*^*-/-*^ mice (Fig 6C). On day 7 p.i., there was approximately a 2-fold increase in CD4^+^CD25^+^Foxp3^+^ T cells in B6 mice and while there was over a 5-fold increase in *Irf8*^*-/-*^ mice. By day 14 p.i., *Irf8*^*-/-*^ mice had approximately twice as many CD4^+^CD25^+^Foxp3^+^ T cells in the MLN as B6 mice. The marked expansion of Tregs in infected *Irf8*^*-/-*^ mice led to significantly higher numbers of CD4^+^CD25^+^Foxp3^+^ T cells in *Irf8*^*-/-*^ than B6 mice, resulting in higher ratios of Foxp3^+^ Tregs to Th2 cells in Hpb-infected *Irf8*^*-/-*^ compared to B6 mice on day 7 as well as day 14 p.i., consistent with the data shown in Fig 3F.

**Fig 7:**
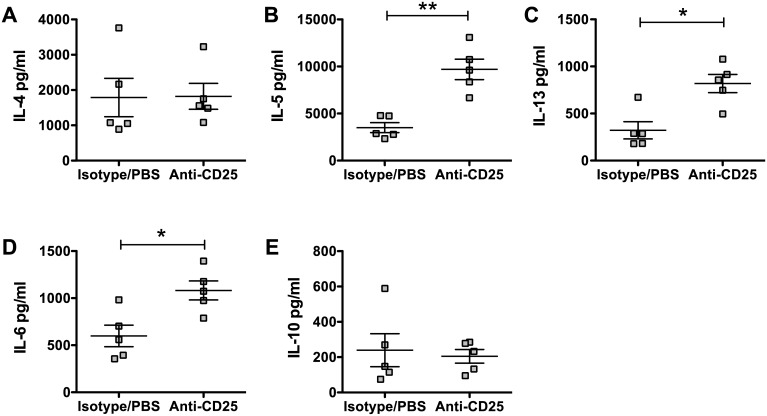
Antigen-specific cytokine secretion by MLN cells from infected *Irf8*^*-/-*^ mice treated with anti-CD25 mAb. Single cell suspensions of MLN were prepared from infected *Irf8*^*-/-*^ mice treated with isotype control antibody or anti-CD25 mAb on day 14 p.i. The cells were stimulated in vitro with 50 μg AWH, supernatants were harvested 48 h later, and cytokine levels were determined using a Bio-Plex assay. (A) IL-4, (B) IL-5, (C) IL-13, (D) IL-6, and (E) IL-10 levels. Data from individual mice are presented as pg/ml and were calculated based on internal standards for each cytokine with the mean ± SEM for each group shown. n = 5 mice per group. *, *p* ≤ 0.05; **, *p* ≤ 0.01.

To investigate if Foxp3^+^ Tregs contributed to high adult worm burdens in *Irf8*^*-/-*^ mice, we treated Hpb-infected *Irf8*^*-/-*^ mice with anti-CD25 mAb or isotype control rat IgG and determined the effects on Th2 responses as well as on the adult worm burden and egg production. Administration of anti-CD25 mAb significantly reduced CD4^+^CD25^+^Foxp3^+^ cells in Hpb-infected *Irf8*^*-/-*^ mice (Fig 6D), while the numbers of CD4^+^GATA3^+^ T cells (Fig 6E), AAMØ (Fig 6F), and MDSC (Fig 6G) were similar to isotype control mice. Depletion of Foxp3^+^ Tregs did not affect the adult worm burden, but egg production was significantly higher in anti-CD25 mAb treated *Irf8*^*-/-*^ mice compared to isotype controls indicating increased worm fecundity (Fig 6H and 6I). We also determined the profile and levels of cytokines secreted ex vivo by MLN cells from anti-CD25 mAb treated and isotype control *Irf8*^*-/-*^ mice in response to AWH. There were significant increases in the Th2 cytokines IL-5 and IL-13 and the pro-inflammatory cytokine IL-6 in the supernatants of infected *Irf8*^*-/-*^ mice treated with anti-CD25 mAb compared to isotype control mice while there were no differences in IL-4, IL-10, or TGFβ (Fig 7 & S6 Fig).

To investigate the effects of depletion of both MDSC and Tregs in IRF-8 deficient mice during Hpb infection, *Irf8*^*-/-*^ mice were treated in tandem with both 5-FU and anti-CD25 mAb as described for single depletions. Control mice were treated with PBS and isotype control antibody. F4/80^-^CD11b^hi^Gr1^hi^ cells as well as CD4^+^CD25^+^Foxp3^+^ cells were significantly reduced in 5-FU and anti-CD25 mAb treated mice (S7A and S7B Fig). Depletion of both MDSC and Tregs in infected *Irf8*^*-/-*^ mice did not affect either the adult worm burden, but egg production was significantly increased similar to mice treated with anti-CD25 mAb alone (S7C and S7D Fig). In contrast to significantly increased levels of IL-5 and IL-13 secreted ex vivo in response to AWH by MLN cells from Treg-depleted compared to control mice shown in Fig 7, serum levels of Th2 cytokines were similar in *Irf8*^*-/-*^ mice treated with anti-CD25 mAb alone or both anti-CD25 mAb and 5-FU compared to control *Irf8*^*-/-*^ mice (S7E–S7G Fig). These date suggest that changes in cytokine levels may vary in supernatants vs. sera of Hpb-infected mice due to differences in kinetics of Th2 cytokine production. Altogether, our data indicate that IRF-8 regulates the expansion of MDSC and Foxp3^+^ Tregs during Hpb infection and modulates Th2 immunity essential for expulsion of adult worms. Depletion of either MDSC or Tregs or both, however, does not completely eliminate the high adult worm burdens in *Irf8*^*-/-*^ mice suggesting that either the depletions were incomplete or that another cell type may be involved.

## Discussion

A partial or complete deficiency in IRF-8 in mice results in increased susceptibility to intracellular pathogens including *T*. *gondii*, *M*. *tuberculosis*, *Salmonella*, and *P*. *chabaudi* AS [36]. Control of these pathogens is dependent on Th1-mediated immune responses, which are deficient in *Irf8*^*-/-*^ and BXH-2 mice. Similar effects have also been reported in a pediatric patient homozygous for a loss-of-function mutation in the IRF8 gene. A deficiency in DC subsets expressing CD8α especially CD11c^+^B220^-^CD8α^+^ DC, the major APC producing IL-12p40 in response to microbial products, is considered to be the underlying defect responsible for the lack of Th1 responses to intracellular pathogens in IRF-8 deficient hosts [12, 17, 37]. Th17 responses are also aberrant in *Irf8*^*-/-*^ mice but the mechanism(s) is only partially understood [18, 19].

In addition to other abnormalities in myeloid-derived cell development, naïve *Irf8*^*-/-*^ mice have a dramatically higher number of MDSC than WT B6 mice [26]. Importantly, significantly fewer MDSC are apparent in tissues and tumors of transgenic mice that over-express *Irf8* compared to WT B6 mice. In addition, *Irf8* expression is down-regulated in MDSC during tumor growth and the frequency of MDSC in patients with breast cancer correlates inversely with IRF-8 levels in these cells [26]. The importance of IRF-8 in regulating MDSC expansion during tumorigenesis is supported by the finding that tumor growth occurs more rapidly in *Irf8*^*-/-*^ than B6 mice.

Based on the findings described above and our previous observation that MDSC increase in B6 mice during Hpb infection, we investigated if IRF-8 contributes to Th2 immunity to infection with a GI nematode [24, 26]. First, we examined *Irf8* expression in MDSC during Hpb infection. *Irf8* expression was significantly down-regulated in MDSC from Hpb-infected compared to naïve B6 mice during primary infection. We also observed that adult worm burdens were significantly higher in mice with a total (*Irf8*^*-/-*^ mice) or partial (BXH-2 mice) loss of functional IRF-8 after both primary and challenge Hpb infections. These data provide compelling evidence that IRF-8 plays an important role in Th2 immunity to Hpb infection.

Analysis of immune cell populations in local and systemic lymphoid tissues from IRF-8-deficient hosts showed significantly higher numbers of total cells and MDSC in *Irf8*^*-/-*^ and BXH-2 compared to B6 mice especially in infected *Irf8*^*-/-*^ mice. Consistent with previous observations, we found significantly higher numbers of MDSC in the MLN as well as the spleen of naïve *Irf8*^*-/-*^ mice [26]. We also observed that naïve BXH-2 have significantly higher numbers of MDSC in the spleen compared to naïve B6 mice. After Hpb infection, MDSC increased in the MLN of *Irf8*^*-/-*^ and BXH-2 mice as we previously observed in B6 mice [24]. There were, however, significantly higher numbers of MDSC in infected *Irf8*^*-/-*^ and BXH-2 mice compared to B6 mice, with *Irf8*^*-/-*^ mice having the largest increase in MDSC among the strains examined. Our findings thus confirm and extend the observations of Waight et al in tumor-bearing mice to IRF-8 deficient mice infected with a GI nematode [26].

Despite marked expansion of MDSC in infected IRF-8 deficient mice, CD4^+^GATA3^+^ T cell numbers were similar in infected *Irf8*^*-/-*^, BXH-2 and B6 mice. Nevertheless, the numbers of AAMØ in MLN and serum levels of HES-specific IgG1 and IgE were significantly lower in association with significantly lower parasite-specific IL-4 secretion ex vivo by MLN cells from infected *Irf8*^*-/-*^ and BXH-2 mice compared to B6 mice. Analysis of co-expression of CD11a and CD49d, considered to be surrogate markers of antigen-experienced CD4^+^ T cells, showed that the frequencies of CD4^+^CD11a^hi^CD49d^hi^ cells increased to similar levels in B6 and *Irf8*^*-/-*^ mice during Hpb infection. The majority of these cells were CD44^hi^CD62L^-^ in infected mice of both genotypes. During infection, 11% of total CD4^+^ T cells in MLN of B6 mice were GATA3^+^ while 12% of total CD4^+^ T cells expressed Foxp3 in infected *Irf8*^*-/-*^ mice. A lower proportion of CD4^+^ T cells from *Irf8*^*-/-*^ mice expressed intracellular IL-4 compared to B6 mice consistent with significantly lower IL-4 secretion ex vivo in response to AWH.

Previous studies by Waight et al demonstrated that purified CD11b^+^Gr1^+^ cells from *Irf8*^*-/-*^ mice strongly inhibit polyclonal as well as allogeneic CD4^+^ T cell proliferation in vitro compared to WT B6 mice [26]. We observed that CD11b^+^Gr1^+^ cells purified from MLN and spleens of naïve and infected *Irf8*^*-/-*^ mice were less potent on a cell-per-cell basis than cells from similar groups of B6 mice in suppressing Con A-induced CD4^+^ T cell proliferation. Previously, we showed that CD11b^+^Gr1^+^ cells from mucosal and systemic tissues of infected B6 mice significantly suppress IL-4 secretion in response to AWH in co-cultures even at a ratio of 1 MDSC:16 responder cells [24]. Similar to differences in the ability to suppress polyclonal CD4^+^ T cell proliferation, CD11b^+^Gr1^+^ cells from infected B6 mice suppressed antigen-specific IL-4 secretion to significantly lower levels than cells from *Irf8*^*-/-*^ mice. Together, these findings suggest the suppressive ability of MDSC from *Irf8*^*-/-*^ mice relative to MDSC from WT B6 mice may vary depending on the experimental model used.

Administration of 5-FU to tumor-bearing mice results in significant decreases in MDSC and enhanced CD8^+^ T cell effector responses [31]. We observed that MDSC were significantly reduced in MLN of 5-FU treated B6 mice on day 7 p.i. In our hands, depletion was transient and the numbers of MDSC rebounded and were significantly increased compared to PBS controls on day 14 p.i. Even so, the adult worm burden was significantly lower in 5-FU treated compared to control B6 mice. This observation is consistent with our previous finding that adoptive transfer of CD11b^+^Gr1^+^ cells from Hpb-infected B6 mice results in a significantly higher worm burden and increased egg production in recipient mice [24]. These data indicate there is an inverse correlation between the number of MDSC and the adult worm burden in line with our findings in the present study in IRF-8 deficient mice. Altogether, these data support the contention that MDSC suppress Th2 immunity to Hpb infection resulting in higher adult worm burdens.

Although 5-FU administration to Hpb-infected *Irf8*^*-/-*^ mice significantly decreased MDSC in the MLN through day 14 p.i., there were no effects on CD4^+^GATA3^+^ T cells, adult worm burden, or egg production. This finding suggested to us that MDSC alone may not be responsible for the increased susceptibility of IRF-8-deficient mice to Hpb infection. Importantly, there were significantly more total CD4^+^ T cells despite similar numbers of CD4^+^GATA3^+^ T cells in MLN of IRF-8-deficient mice compared to B6 mice after Hpb infection. This difference was due to a significantly higher number of CD4^+^CD25^+^Foxp3^+^ cells in infected *Irf8*^*-/-*^ than B6 mice. Indeed, CD4^+^CD25^+^Foxp3^+^ Tregs constituted a larger proportion of the total CD4^+^ T cells in MLN of infected *Irf8*^*-/-*^ than B6 mice, resulting in a higher ratio of this T cell population to CD4^+^GATA3^+^ Th2 cells in *Irf8*^*-/-*^ mice.

Previous studies indicate that Foxp3^+^ Tregs expand in local lymphoid tissues, including the lamina propria, Peyer’s patches, and MLN, as well as systemically in the spleen of Hpb-infected B6 and BALB/c mice [34, 35, 38]. Although Foxp3^+^ Tregs contribute to suppressed Th2 cytokine responses during Hpb infection, their effect on adult worm expulsion is variable [35, 39]. Several strategies have been used to increase Foxp3^+^ Tregs or to partially or completely deplete these cells in infected BALB/c and B6 mice [35, 39]. Together, the results of these studies provide evidence of a complex role for Foxp3^+^ Tregs in immunity to Hpb infection. The overall effect of increasing Foxp3^+^ Tregs or depleting these cells differs not only on the genetic background of the host but also on the depletion strategy used. Recently, it was proposed that a low number of Foxp3^+^ Tregs may be beneficial in providing a balance between a protective Th2 response and harmful pro-inflammatory response [35].

In the present study, we depleted Foxp3^+^ Tregs in infected *Irf8*^*-/-*^ mice using repeated treatments with an anti-CD25 mAb prior to infection, on the day of infection and day 7 p.i. Consistent with previous studies, there were significant increases in Th2 cytokines as well as IL-6 compared to isotype control, infected *Irf8*^*-/-*^ mice, but there were no effects on the adult worm burden or the numbers of CD4^+^GATA3^+^ T cells or AAMØ [35, 38, 39]. Interestingly, egg production was significantly increased in Treg-depleted compared to isotype control *Irf8*^*-/-*^ mice indicating increased worm fecundity in the depleted mice, but the basis of the increase is not clear. Depletion of both MDSC and Foxp3^+^ Tregs using 5-FU and anti-CD25 mAb did not affect serum levels of Th2 cytokines or the adult worm burden in Hpb-infected *Irf8*^*-/-*^ mice, but the number of eggs/g feces was significantly increased.

In conclusion, our data demonstrate that a high adult worm burden in Hpb-infected IRF-8 deficient mice is associated with decreased AWH-specific IL-4 secretion by MLN cells ex vivo, fewer AAMØ, and marked expansion of MDSC in MLN. Compared to infected B6 mice, *Irf8*^*-/-*^ mice had significantly lower serum levels of HES-specific IgG1 and IgE and significantly higher numbers of CD4^+^ T cells and Foxp3^+^ Tregs in MLN during Hpb infection. Although MDSC as well as Foxp3^+^ Tregs suppress critical Th2 responses, depleting these cell populations individually or in tandem did not promote adult worm expulsion in infected *Irf8*^*-/-*^ mice. This may be due to the limitations of using 5-FU and anti-CD25 mAb to deplete MDSC and Foxp3 Tregs, respectively [31, 40]. Nevertheless, our data provide novel information supporting a role for IRF-8 in the development of Th2 immunity to a GI nematode infection.

## Materials and methods

### Ethics statement

The animal studies reported in this paper were approved and conducted in accordance with the guidelines and recommendations of The Animal Care and Use Committee of McGill University (#2015–7592). Animals were sacrificed by CO_2_ overdose under isoflurane anesthesia.

### Mice, parasite and infection

Male and female B6 mice, 8–10 weeks old, were purchased from Charles River Laboratories (St. Constant, QC). *Irf8*^-/-^ mice, generated as previously described, were bred and maintained at the Research Institute of the McGill University Health Centre from breeding stock generously provided by Dr. Keiko Ozato (NICHD, NIH) [7]. BXH-2 mice were obtained from the colony maintained by Dr. P. Gros at McGill University [13]. Hpb was maintained and propagated as described previously [41]. Mice were age- and sex-matched for all experiments. For primary infection, mice were infected per os (p.o.) with 200 infective third-stage Hpb larvae (L3). To determine egg production, fecal pellets were collected on day 13 p.i. and eggs were enumerated by the McMaster method [41]. To assess parasite burden, mice were sacrificed on day 14 p.i., the intestines harvested, and the number of adult worms in each mouse was counted by dissection microscopy. For challenge infection, mice were infected as described above and treated p.o. with a single dose of pyrantel pamoate (Combantrin; Pfizer Canada, Montreal, QC) at 100 mg/kg body weight on day 14 p.i. to eliminate adult worms. Drug-cured mice were allowed to rest for 5 weeks and then re-infected p.o. with 200 L3. Adult worm homogenate (AWH) was prepared as described previously [41], the protein concentration determined using a Bradford protein assay kit (Bio-Rad, Hercules, CA), and stored at -20°C until use.

### Cell preparation

MLN and spleens were harvested from naïve and Hpb-infected mice on the indicated days p.i. Briefly, single cell suspensions were prepared using a sterile wire mesh. The cells were centrifuged, re-suspended in 0.175 M NH_4_Cl to lyse red blood cells, washed, and re-suspended in RPMI 1640 medium (Life Technologies, Burlington, ON, Canada) supplemented with 5% heat-inactivated fetal calf serum (HyClone Laboratories, Logan, UT), 25 mM HEPES (Life Technologies), 0.12% gentamicin (Life Technologies), and 2 mM glutamine (Life Technologies) (complete medium). Dead cells were removed using a dead cell removal kit from Miltenyi Biotec (Auburn, CA) according to the manufacturer’s instructions. Immune cell populations were purified using antibody-conjugated magnetic beads according to the manufacturer’s instructions (Miltenyi Biotec). To obtain CD11b^+^Gr1^+^ cells, MLN and spleen cells were enriched by negative selection of CD11c^+^ cells using anti-CD11c-conjugated beads followed by positive selection of CD11b^+^ cells using anti-CD11b-conjugated beads. The resultant cell population had a purity of >96% for CD11c^-^CD11b^+^Gr1^+^ cells as determined by flow cytometry.

To determine cytokine secretion in response to AWH, single cell suspensions of MLN cells were adjusted to 5 x 10^6^ cells/ml in complete medium. Aliquots of 5 x 10^6^ cells were added to 48-well tissue culture plates and stimulated in triplicates with medium or 50 μg/ml AWH. The cultures were incubated at 37°C in 5% CO_2_ for 48 h, and supernatants were collected for cytokine determination by ELISA or multiplex assay.

### *In vitro* suppression assays

To assess suppression of Con A-induced proliferation, MLN and spleens were harvested from naïve and infected B6 or *Irf8*^*-/-*^ mice on day 7 p.i. CD11b^+^Gr1^+^ cells were purified as described above and co-cultured with spleen cells from naïve B6 mice labeled with CFSE (Life Technologies) as previously described [41]. Aliquots of 1 x 10^6^ CFSE-labeled spleen cells in complete RPMI 1640 medium were added to 96-well plates and CD11b^+^Gr1^+^ cells were added at the indicated ratios. The co-cultures were stimulated with 2 μg/ml Con A (Sigma, St. Louis, MO) for 72 h at 37°C in 5% CO_2_, and the cells were harvested and stained with APC-conjugated anti-CD4 mAb (clone GK1.5; eBioscience, San Diego, CA). CD4^+^ T cells were gated, the CFSE dilution analyzed by flow cytometry, and cell cycle progression was determined using FlowJo software (FlowJo LLC, Ashland, OR). Data are presented as the percentage of total CD4^+^ T cells that proliferated. For suppression of IL-4 secretion in response to AWH, CD11b^+^Gr1^+^ cells purified from MLN or spleen of naive and infected B6 or *Irf8*^*-/-*^ mice on day 7 p.i. were co-cultured in complete medium at the indicated ratios with 1 x 10^6^ spleen cells obtained from Hpb-infected B6 mice on day 14 p.i. The cultures were stimulated with 50 μg/ml AWH for 48 h, the supernatants were harvested, and IL-4 levels were determined by ELISA.

### Immunophenotyping

Single cell suspensions of MLN and spleen were prepared as described above. Aliquots of 1–2 x 10^6^ cells were stained with viability dye (eFluor 780 or eFluor 506; eBioscience, San Diego, CA), and Fc receptors blocked with anti-mouse CD16/CD32 (clone 2.4G2; BD Biosciences, San Jose, CA). To identify MDSC and AAMØ, cells were stained with PE-conjugated anti-F4/80 (clone BMB; eBioscience), APC-conjugated anti-CD11b (clone M1/70; eBioscience), and PerCP-Cy5.5-conjugated anti-Gr1 (clone RB6-8C5; eBioscience) mAbs. Surfaced stained cells were fixed using a fixation and permeabilization kit (eBioscience) prior to intracellular staining with FITC-conjugated anti-CD206 mAb (clone C068C2; BioLegend). To identify Th cell subsets, cells were surface-stained with FITC-conjugated anti-CD4 mAb (clone GK1.5; eBioscience), fixed and permeabilized, and stained intracellularly with PE-conjugated anti-GATA3 (clone TWAJ; eBioscience), PE-conjugated anti-Foxp3 (Clone FJK-16a; eBioscience), or PE-conjugated anti-RORγt (clone B2D; eBioscience) mAb. In some experiments, cells were surface stained with the following antibodies from BioLegend: FITC-conjugated anti-CD4 (Clone GK1.5), PerCP/Cy5.5-conjugated anti-CD11a (Clone M17/4), PE-conjugated anti-CD49d (Clone 9C10), APC-conjugated anti-CD44 (Clone 1M7), and BV421-conjugated anti-CD62L (Clone MEL-14). For intracellular cytokine staining (ICS), cells were stimulated in vitro with PMA (50 ηg/ml) and ionomycin (1 μg/ml) in the presence of brefeldin A (5 μg/ml) and incubated at 37°C in 5% CO_2_ for 5 h. Stimulated cells were stained with viability dye followed by anti-mouse CD16/CD32 to block Fc receptors and surface stained with FITC-conjugated anti-CD4 (Clone GK1.5, eBioscience) mAb. The cells were fixed, permeabilized, and stained intracellularly with APC-conjugated anti-IL-4 (Clone 11B11; eBioscience), PE-conjugated anti-IFN-γ (Clone XMG1.2; eBioscience), or APC-conjugated anti-IL-17 (Clone TC11.18H10.1; eBioscience) mAb. UltraComp eBeads (eBioscience) were stained with each fluorochrome in the panel of markers as compensation controls. Flow cytometry was performed using a LSR Fortessa (BD Biosciences) and the data were analyzed using FlowJo software (FlowJo, LLC).

### qRT-PCR

To analyze *Irf8* expression in tissue, MLN and spleen were harvested from naïve and infected B6 mice on day 7 p.i and immediately snap-frozen in liquid nitrogen. Frozen tissues were stored at -80°C until processing. To analyze *Irf8* expression in MDSC, MLN and spleen were harvested from separate groups of naïve and infected B6 mice on day 7 p.i. Single cell suspensions were prepared from tissues of individual mice and CD11c^-^CD11b^+^Gr1^+^ cells were purified as described above. Even with pooling, insufficient CD11b^+^Gr1^+^ cells were obtained from MLN of naïve or infected mice for analysis of *Irf8* expression by qRT-PCR. To obtain 2 x 10^6^ MDSC for analysis, CD11b^+^Gr1^+^ cells purified from the spleens of two naïve B6 mice were pooled while 2 x 10^6^ CD11b^+^Gr1^+^ cells from the spleen of individual infected mice were analyzed separately.

Total RNA was extracted from frozen tissues and purified CD11b^+^Gr1^+^ cells using TRIzol reagent (Life Technologies) according to the manufacturer’s instructions. RNA integrity was confirmed by denaturing agarose gel electrophoresis, followed by staining with ethidium bromide, and visualized on a UV trans-illuminator. Purity and quantification of RNA were determined by UV absorbance ratio of A260/A280. First strand cDNA synthesis was performed using 1 μg purified RNA in 20 μl reaction using iScript reverse transcriptase kit (Bio-Rad, Hercules, CA) according to the manufacturer’s instructions. A no-enzyme RT control was also prepared for each sample. First strand synthesis products of all samples including the no-RT controls were analyzed immediately by qRT-PCR using TaqMan probe-based gene expression assays (Applied Biosystems, Waltham, MA) for *Irf8* (assay ID Mn00492567_m1) and *Actb* (beta-actin, assay ID Mn00607939_s1) as an endogenous control. qRT-PCR reactions in 20 μl in triplicates were prepared in a MicroAmp optical 96-well plate (Applied Biosystems) using 1 ηg cDNA and Taqman Universal PCR Master Mix (Applied Biosystems). No template controls were also included for each gene assayed on the plate. Reactions were performed in a StepOne real-time PCR instrument (Applied Biosystems) using a pre-set, standard run thermal cycling condition of 40 cycles. Data were analyzed using the StepOne software (Applied Biosystems). Relative quantification of gene expression was determined using the comparative Ct (ΔΔCt) method.

### In vivo depletion of MDSC

For in vivo depletion of MDSC, B6 and *Irf8*^*-/-*^ mice were treated i.p. with 200 μl 5-FU (Sigma, St. Louis, MO) at a dose of 50 mg/kg on day -2 before infection and days 5 and 12 p.i. [31]. Control mice were injected i.p. with 200 μl PBS on the days corresponding to 5-FU treatment. The extent of MDSC depletion was verified in MLN cells obtained from 5-FU treated and PBS control mice on day 14 p.i. by flow cytometry as described for immunophenotyping.

### In vivo depletion of CD25^+^ cells

To deplete Tregs, *Irf8*^*-/-*^ mice were treated i.p. with 1 mg of neutralizing anti-CD25 mAb (clone PC61, rat IgG1) or control rat IgG (isotype control) (Bio X Cell; West Lebanon, NH) on day -7 before infection, day 0, and day 7 p.i. [40]. The extent of Treg depletion was verified in MLN cells obtained from anti-CD25 mAb treated and isotype control mice on day 14 p.i. by flow cytometry. Single cell suspensions were prepared and the cells were surfaced stained with FITC-conjugated anti-CD4 mAb (clone GK1.5; eBioscience) and PE-conjugated anti-CD25 mAb (clone 7D4; eBiosciences), fixed, and permeabilized followed by intracellular staining with APC-conjugated anti-Foxp3 mAb (clone FJK-16s; eBioscience).

### Cytokine determination

IL-4 levels in culture supernatants were determined by a sandwich ELISA specific for murine IL-4 using paired capture and detection antibodies (clone 11B11 and biotin-conjugated clone BVD6-24G2; eBioscience) and developed by streptavidin-HRP conjugate (R&D Systems, Minneapolis, MN) and ABTS substrate (Roche, Indianapolis, IN) as previously described [42]. Cytokine concentrations were calculated based on a standard curve generated using serial dilutions of murine recombinant IL-4 (R&D Systems). In some experiments, cytokine levels were determined in supernatants of AWH-stimulated MLN cells or serum using a custom-made cytokine Bio-Plex Pro 10-Plex assay (Bio-Rad, Hercules CA) according to the manufacturer’s instructions. Acquisition was performed on the MAGPIX platform (Luminex) and data were analyzed using the Bio-Plex Manager 6.1 software (Bio-Rad). To determine TGFβ levels, supernatants were acid-activated and neutralized prior to assay using an ELISA performed according to the manufacturer’s instructions (Mouse TGFβ DuoSet, R&D Systems).

### Statistical analyses

Data are presented as mean ± SEM. Groups were compared and differences were analyzed for significance using an unpaired Student’s *t*-test with Welsh’s correction. For multiple groups, differences were analyzed for significance using one-way ANOVA followed by Bonferroni post-hoc test. Prism software (GraphPad Software, La Jolla CA) was used for all statistical analyses. A *p* value <0.05 was considered statistically significant.

## Supporting information

S1 Fig *Irf8* expression in MLN and spleen of naïve and Hpb-infected C57BL/6 (B6) mice. (PDF)

S2 Fig Cellularity of MLN and spleen of naïve and Hpb-infected C57BL/6 (B6) and IRF-8 deficient mice. (PDF)

S3 Fig Total F4/80^-^CD11b^hi^Gr1^hi^ cells in naïve C57BL/6 (B6) and IRF-8 deficient mice and immunophenotype and IL-4 secretion by MLN cells from Hpb-infected B6 and BXH-2 mice. (PDF)

S4 Fig Frequencies of MDSC in MLN of naïve and Hpb-infected C57BL/6 (B6) or IRF-8 deficient mice. (PDF)

S5 Fig CD4^+^ T cell proliferation is suppressed by splenic CD11b^+^Gr1^+^ cells from naïve or Hpb-infected C57BL/6 and *Irf8*^*-/-*^ mice. (PDF)

S6 Fig TGFβ levels in the supernatants of MLN cells stimulated ex vivo with AWH. (PDF)

S7 Fig Effects of MDSC and Treg depletion in Hpb-infected *Irf8*^*-/-*^ mice on Th2 immune responses and infection outcome. (PDF)
