# Supplementary material for: IRF-8 regulates expansion of myeloid-derived suppressor cells and Foxp3+ regulatory T cells and modulates Th2 immune responses to gastrointestinal nematode infection
Source: PLoS Pathog. 2017 Oct 2;13(10):e1006647. doi: 10.1371/journal.ppat.1006647 (PMC5638610; doi:10.1371/journal.ppat.1006647)

S1 Fig. I*rf8* expression in MLN and spleen of naïve and Hpb-infected C57BL/6 (B6) mice. *Irf8* expression was determined by qRT-PCR in the MLN and spleen of naïve and infected B6 mice on day 7 p.i. Each point represents an individual mouse. Data are presented as relative quantity of *Irf8* normalized against the endogenous control *Actb*. Data are presented as mean ± SEM.

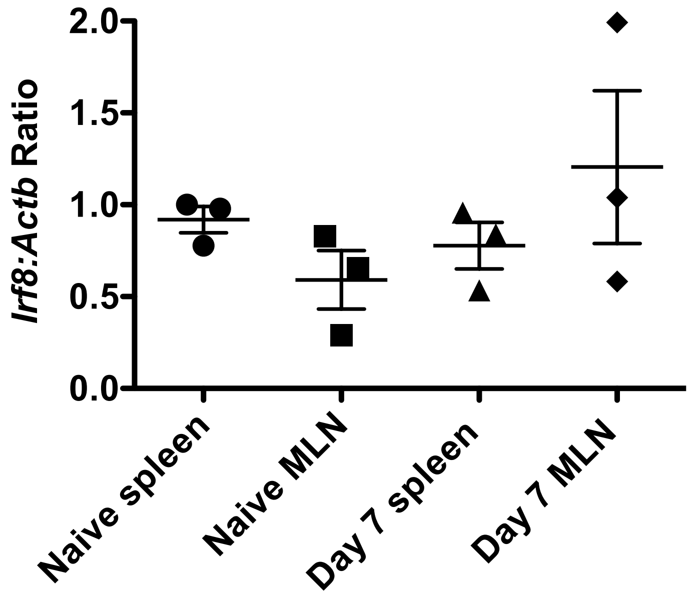

Supplement: S1 Fig — (PDF) [file ppat.1006647.s001.pdf]
