# Supplementary material for: IRF-8 regulates expansion of myeloid-derived suppressor cells and Foxp3+ regulatory T cells and modulates Th2 immune responses to gastrointestinal nematode infection
Source: PLoS Pathog. 2017 Oct 2;13(10):e1006647. doi: 10.1371/journal.ppat.1006647 (PMC5638610; doi:10.1371/journal.ppat.1006647)

S2 Fig. Cellularity of MLN and spleen of naïve and Hpb-infected C57BL/6 (B6) and IRF-8 deficient mice. Total cell numbers were determined in (A) MLN and (B) spleen of naïve and infected B6, BXH-2, or *Irf8*$^{-/-}$ mice on day 14 p.i. after primary or challenge Hpb infection. n=7-15 mice per group. Data pooled from independent experiments are shown and are presented as mean ± SEM. *, p ≤ 0.05; ****, p ≤ 0.0001.

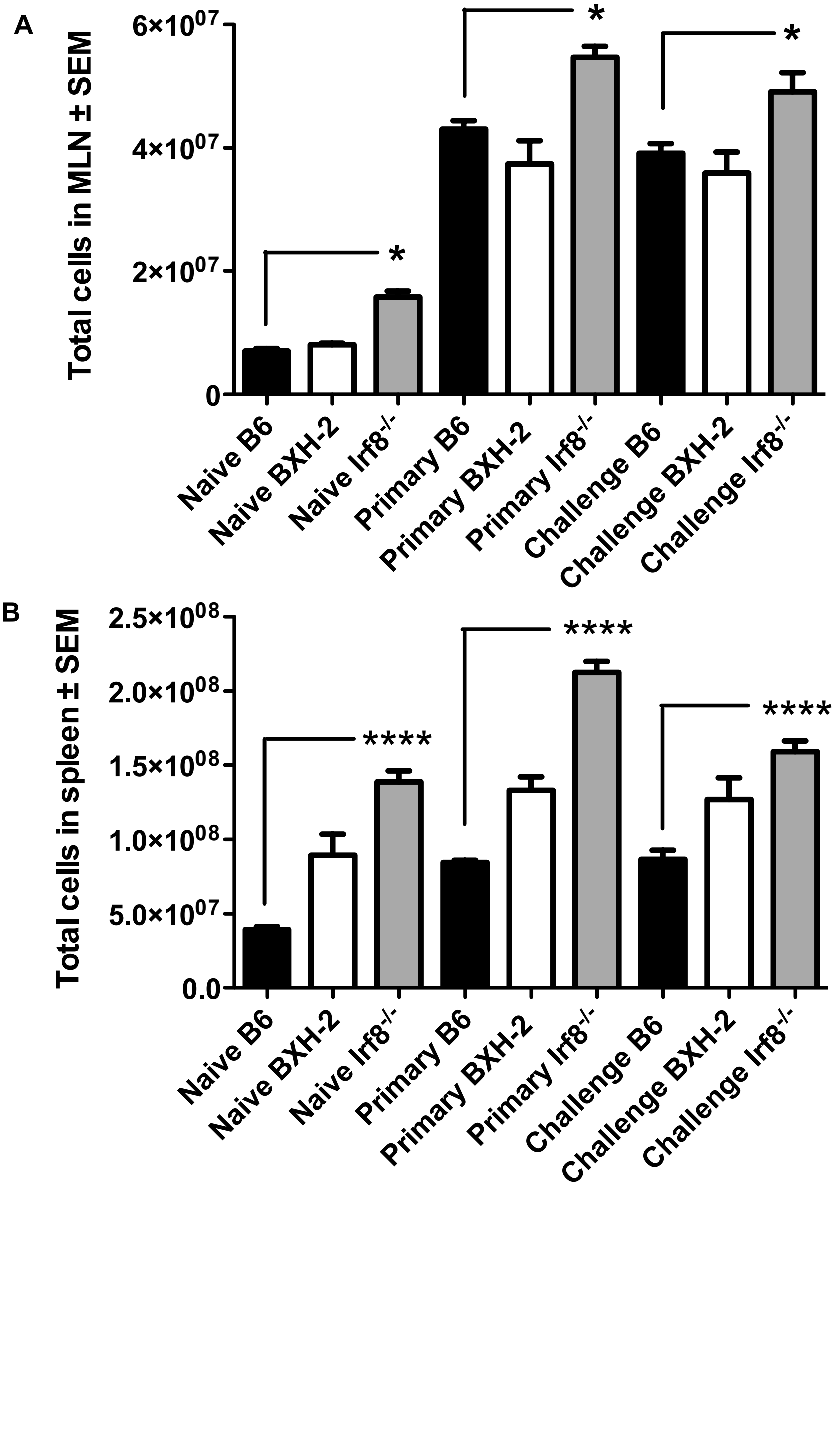

Supplement: S2 Fig — (PDF) [file ppat.1006647.s002.pdf]
