# Supplementary material for: IRF-8 regulates expansion of myeloid-derived suppressor cells and Foxp3+ regulatory T cells and modulates Th2 immune responses to gastrointestinal nematode infection
Source: PLoS Pathog. 2017 Oct 2;13(10):e1006647. doi: 10.1371/journal.ppat.1006647 (PMC5638610; doi:10.1371/journal.ppat.1006647)

**S3 Fig. Total $F4/80^{-}CD11b^{hi}Gr1^{hi}$ cells in naïve C57BL/6 (B6) and IRF-8 deficient mice and immunophenotype and IL-4 secretion by MLN cells from Hpb-infected B6 and BXH-2 mice.** Total numbers of $F4/80^{-}CD11b^{hi}Gr1^{hi}$ cells (MDSC) in MLN and spleen of naïve (A) C57BL/6 and *Irf8*$^{-/-}$ and (B) C57BL/6 and BXH-2 mice. (C) Total numbers of $CD4^{+}GATA3^{+}$ T cells and (D) $F4/80^{+}CD11b^{+}CD206^{+}$ cells (AAMØ) in MLN of infected C57BL/6 and BXH-2 mice on day 14 p.i. (E) AWH-induced IL-4 secretion ex vivo by MLN cells from Hpb-infected C57BL/6 and BXH-2 mice on day 14 p.i. The cells were stimulated with medium or 50 µg AWH, the supernatants were collected 48 h later, and IL-4 levels were determined by ELISA. Data are representative of three replicate experiments for C57BL/6 and *Irf8*$^{-/-}$ mice and two replicate experiments for C57BL/6 and BXH-2 mice (n = 5 mice per group). Data are presented as mean ± SEM. ND, not detectable; ns, not significant; **, p≤0.01, ***, p≤0.001; ****, p≤0.0001.

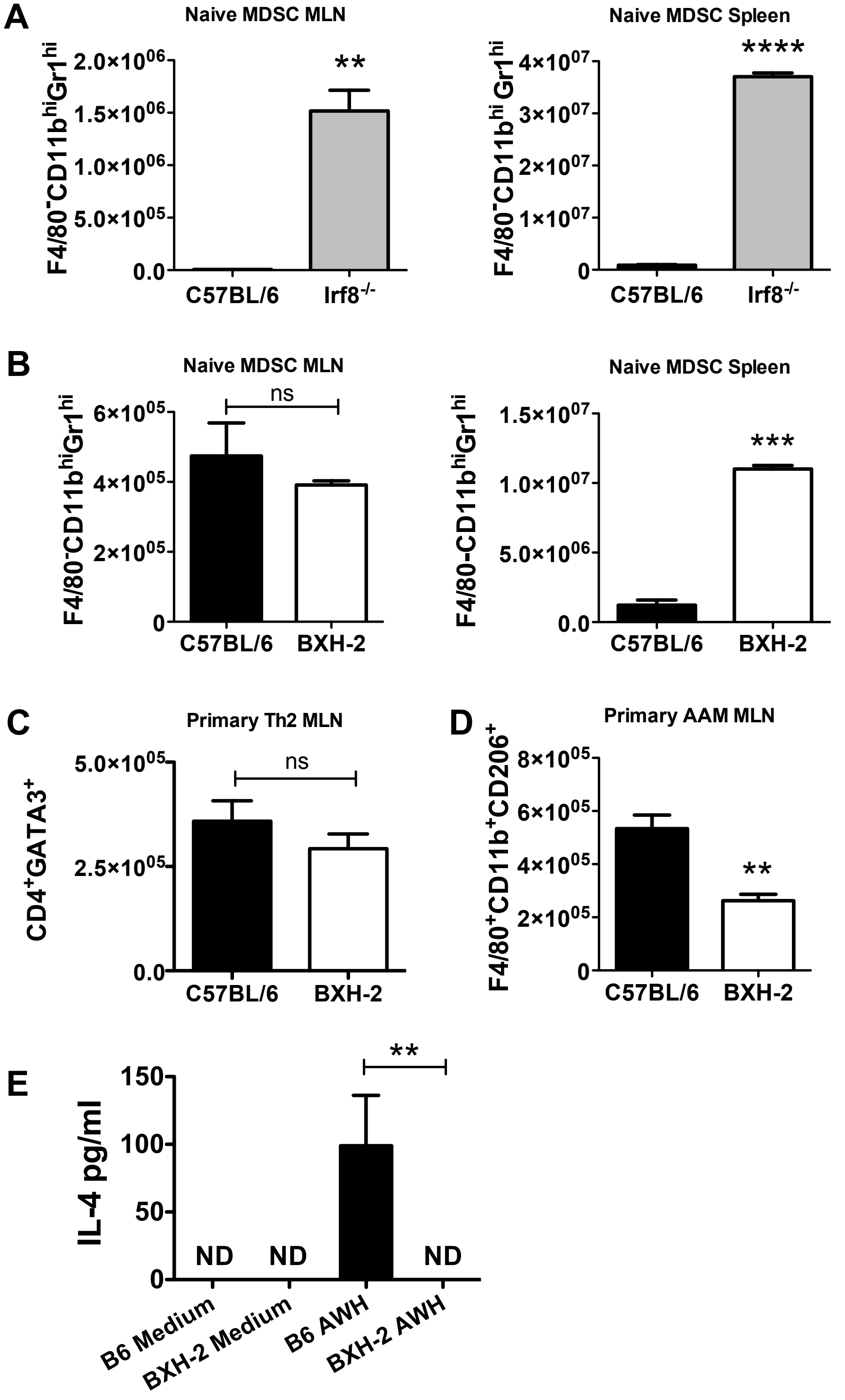

Supplement: S3 Fig — (PDF) [file ppat.1006647.s003.pdf]
