# Supplementary material for: IRF-8 regulates expansion of myeloid-derived suppressor cells and Foxp3+ regulatory T cells and modulates Th2 immune responses to gastrointestinal nematode infection
Source: PLoS Pathog. 2017 Oct 2;13(10):e1006647. doi: 10.1371/journal.ppat.1006647 (PMC5638610; doi:10.1371/journal.ppat.1006647)

S4 Fig. Frequencies of MDSC in MLN of naïve and Hpb-infected C57BL/6 (B6) or IRF-8 deficient mice. The frequencies of F4/80$^-$CD11b^{hi}Gr1^{hi} cells (MDSC) were determined in MLN of naïve and Hpb-infected B6, *Irf8*$^{-/-}$ and BXH-2 mice on day 14 p.i. by flow cytometry using the gating strategy shown in Fig 2A. Data are representative of three replicate experiments for B6 and I*rf8*$^{-/-}$ mice and two replicate experiments for BXH-2 mice (n=5 mice per group). Data are presented as mean ± SEM. *, p≤0.05; **, p≤0.01; ***, p≤0.001 compared to naïve B6 mice; ####, p≤0.0001 compared to naïve *Irf8*$^{-/-}$ mice; ns, not significant compared to naïve BXH-2 mice.

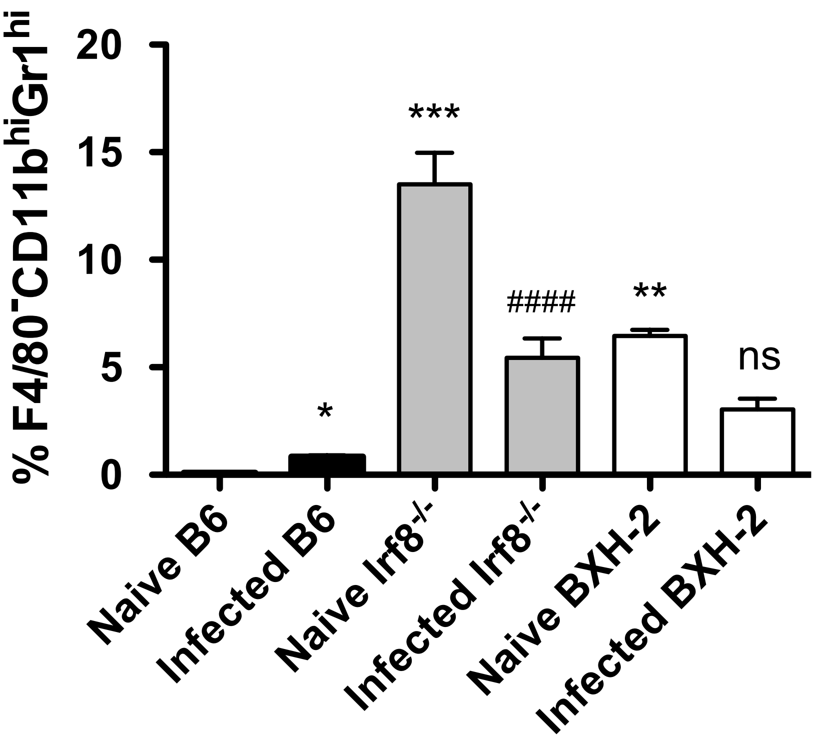

Supplement: S4 Fig — (PDF) [file ppat.1006647.s004.pdf]
