# Supplementary material for: IRF-8 regulates expansion of myeloid-derived suppressor cells and Foxp3+ regulatory T cells and modulates Th2 immune responses to gastrointestinal nematode infection
Source: PLoS Pathog. 2017 Oct 2;13(10):e1006647. doi: 10.1371/journal.ppat.1006647 (PMC5638610; doi:10.1371/journal.ppat.1006647)

S5 Fig. CD4+ T cell proliferation is suppressed by splenic CD11b+Gr1+ cells from naïve or Hpb-infected C57BL/6 and *Irf8*-/- mice. CD11b+Gr1+ cells purified from the spleen of naïve or Hpb-infected mice on day 7 p.i. were co-cultured with CFSE-labeled spleen cells from naïve C57BL/6 mice at a ratio of 1:8 (MDSC:responder cells). The cultures were stimulated with 2 µg/ml Con A and 72 h later, CFSE dilution was analyzed in gated CD4+ T cells by flow cytometry. Positive and negative controls are shown in Fig 4D.

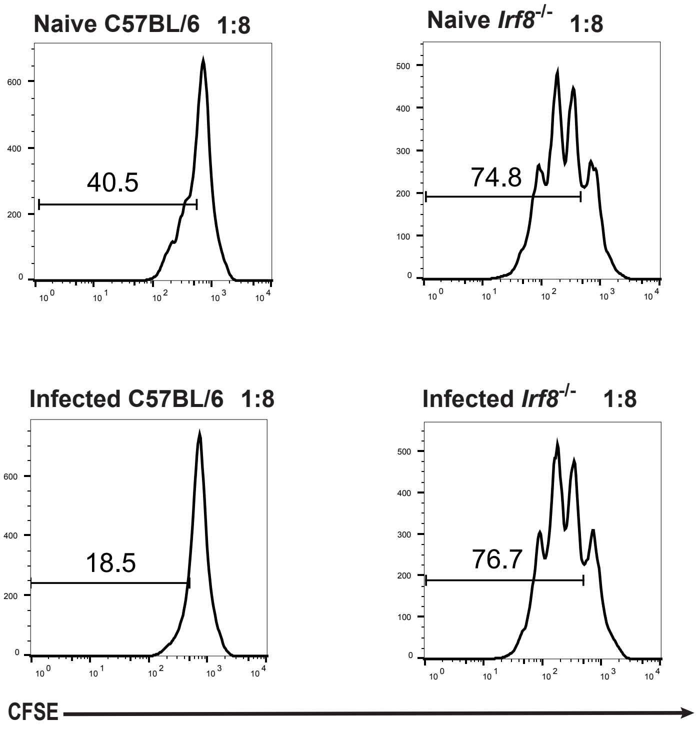

Supplement: S5 Fig — (PDF) [file ppat.1006647.s005.pdf]
