# Supplementary material for: IRF-8 regulates expansion of myeloid-derived suppressor cells and Foxp3+ regulatory T cells and modulates Th2 immune responses to gastrointestinal nematode infection
Source: PLoS Pathog. 2017 Oct 2;13(10):e1006647. doi: 10.1371/journal.ppat.1006647 (PMC5638610; doi:10.1371/journal.ppat.1006647)

S6 Fig. TGFβ levels in the supernatants of MLN cells stimulated ex vivo with AWH. TGFβ levels were determined by ELISA in the supernatants of AWH-stimulated MLN cells from *Irf8*$^{-/-}$ mice treated with isotype control antibody or anti-CD25 mAb as described in the Materials and Methods. Each point represents an individual mouse. Data are presented as mean ± SEM.

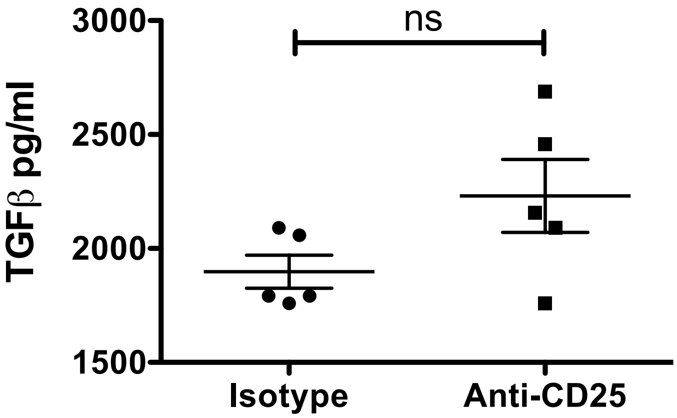

Supplement: S6 Fig — (PDF) [file ppat.1006647.s006.pdf]
