# Supplementary material for: IRF-8 regulates expansion of myeloid-derived suppressor cells and Foxp3+ regulatory T cells and modulates Th2 immune responses to gastrointestinal nematode infection
Source: PLoS Pathog. 2017 Oct 2;13(10):e1006647. doi: 10.1371/journal.ppat.1006647 (PMC5638610; doi:10.1371/journal.ppat.1006647)

S7 Fig. Effects of MDSC and Treg depletion in Hpb-infected *Irf8*$^{-/-}$ mice on Th2 immune responses and infection outcome. Hpb-infected *Irf8*$^{-/-}$ mice were treated with 5-FU and anti-CD25 mAb or PBS and isotype antibody as controls as described for single depletion of MDSC or Tregs in Materials and Methods. On day 14 p.i., MLN were harvested for immunophenotying and sera were collected for determination of cytokines by Bio-plex assay. Total numbers of (A) F4/80^{-}CD11b^{hi}Gr1^{hi} cells (MDSC) and (B) CD4^{+}CD25^{+}Foxp3^{+} Tregs in MLN of treated and control Hpb-infected *Irf8*$^{-/-}$ mice on day 14 p.i., (C) adult worm burdens, (D) egg production, and serum levels of (E) IL-4, (F) IL-5 and (G) IL-13. Each point represents an individual mouse (n=4-5 mice per group). Cytokine levels are presented as pg/ml and were calculated based on internal standards for each cytokine. Data are presented as mean ± SEM. ns, not significant; *, p≤0.05, **, p≤0.01; ***, p≤0.001.

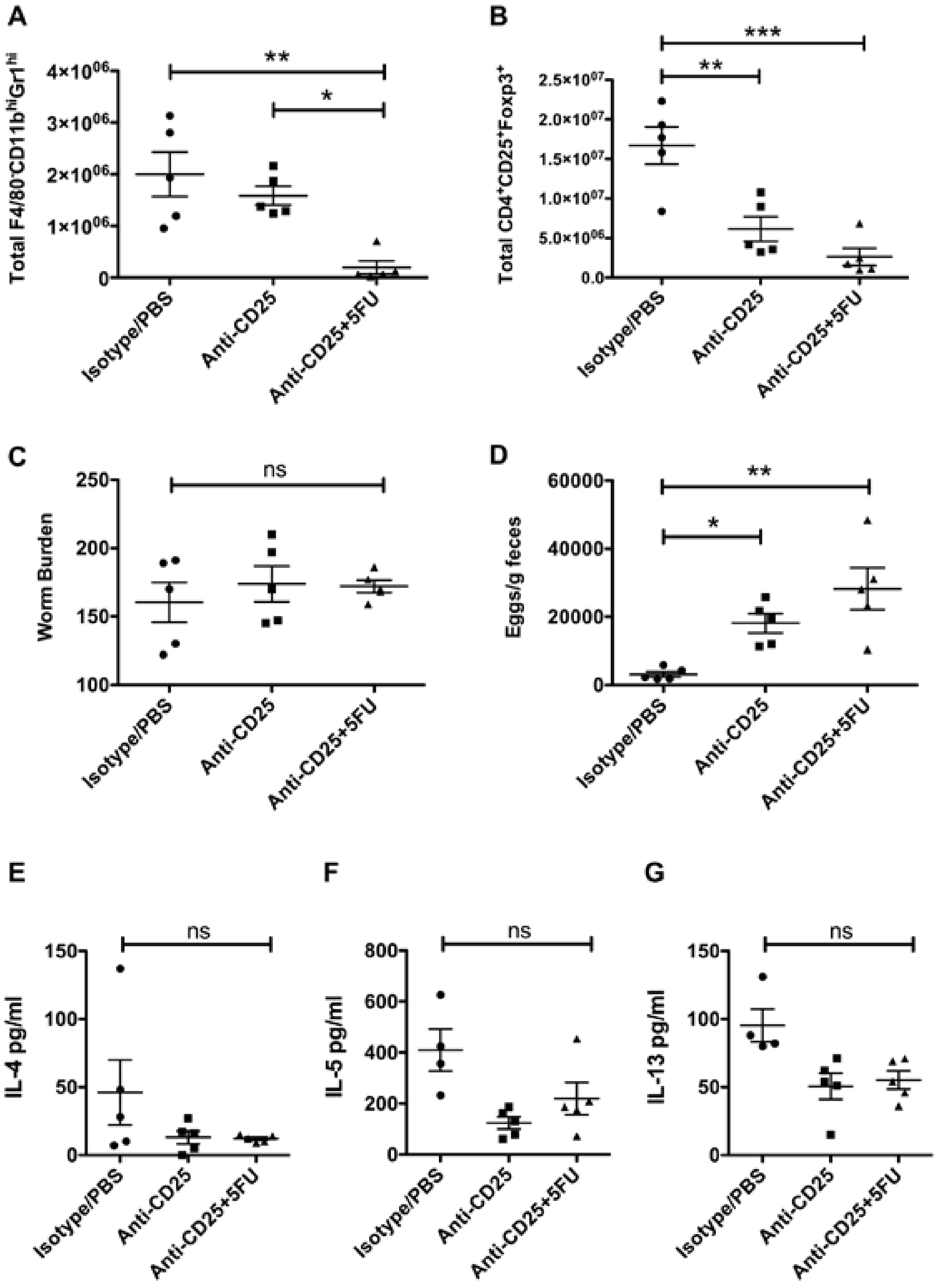

Supplement: S7 Fig — (PDF) [file ppat.1006647.s007.pdf]
